# Exome sequencing identified new mutations in a Marfan syndrome family

**DOI:** 10.1186/1746-1596-9-25

**Published:** 2014-01-31

**Authors:** Guangxin Li, Jian Yu, Kun Wang, Bin Wang, Minghai Wang, Shuguang Zhang, Shiyong Qin, Zhenhai Yu

**Affiliations:** 1 Department of Vascular Surgery, Qianfoshan Hospital, No.16766 Jingshi Road, Jinan 250014, Shandong, China; 2 Department of Hepatobiliary Surgery, Qilu Hospital of Shandong University, Jinan 250012, Shandong, China

**Keywords:** Exome sequencing, New mutations, Marfan syndrome, *FBN1*, *RP1*

## Abstract

Marfan syndrome is a common autosomal dominant hereditary connective tissue disorder. There is no cure for Marfan syndrome currently. Next-generation sequencing (NGS) technology is efficient to identify genetic lesions at the exome level. Here we carried out exome sequencing of two Marfan syndrome patients. Further Sanger sequencing validation in other five members from the same family was also implemented to confirm new variants which may contribute to the pathogenesis of the disease. Two new variants, including one nonsense SNP in the Marfan syndrome gene *FBN1* and one missense mutation in exon 15 of *LRP1*, which may be related to the phenotype of the patients were identified. The exome sequencing analysis provides us a new insight into the molecular events governing pathogenesis of Marfan syndrome.

**Virtual slide:**

http://www.diagnosticpathology.diagnomx.eu/vs/1229110069114125.

## Introduction

Marfan syndrome is a common autosomal dominant hereditary connective tissue disorder with prominent manifestations in different organ systems, including cardiovascular, ocular, and skeletal system [1]. Globally, about 1 in 5000 to 1 in 10,000 live newborns is affected without any racial, geographical or occupational predilection [1-3]. The Marfan syndrome gene, *FBN1*, was localized on chromosome 15q21 and cloned in 1991 [4]. Numerous therapy strategies have been proposed ever since. However, the mortality remains high and there is no cure for Marfan syndrome currently. This is mainly because the understanding of the underlying mechanism is still limited. Identify new genetic lesions of the disease may result in ideas which present alternatives of up to now inadequate therapy strategies.

Next-generation sequencing (NGS) technologies is efficient to identify genetic lesions at the exome level [5], especially for families that are not big enough for classical linkage studies. Studies on familial thoracic aortic disease [6] or other syndromes [7,8] which presents malformations overlapped with Marfan syndrome have identified new mutations which is likely responsible for the clinical phenotype. However, there is no exome-sequencing study specific for Marfan syndrome currently.

Here we carried out exome sequencing of two Marfan syndrome patients. Further Sanger sequencing validation in other five members from the same family was also implemented to confirm new variants which may contribute to the pathogenesis of the disease. Our investigation here may provide new insights for the molecular mechanism of Marfan syndrome.

## Materials and methods

### Sample and DNA preparation

**Figure 1:**
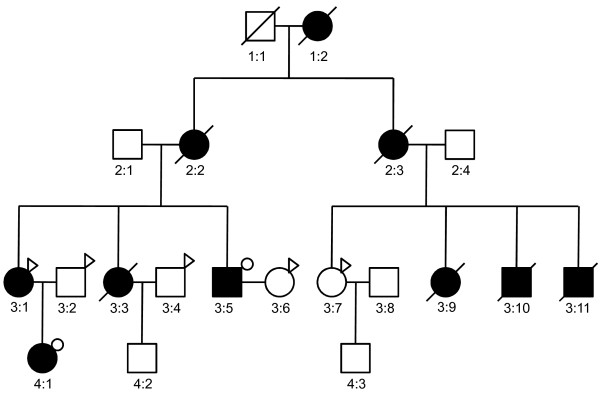
**Pedigree of Marfan syndrome family.** Circles represent female participants and squares male participants. A slash through the symbol indicates that the family member is deceased. Black symbols indicate patients with Marfan syndrome. Small circles on the top right indicate members for whom whole-exome sequencing and Sanger sequencing validation were carried out. Arrows on the top right indicate members for whom the PCR and Sanger sequencing validation were carried out.

The Institutional Review Board (IRB) at the Qianfoshan hospital approved the study. Prior to their participation, written informed documents were reviewed and obtained from all subjects. Pedigree of the family is shown in Figure 1. Peripheral blood was collected and genomic DNA was isolated from current available cases (3:1, 4:1, and 3:5) and several unaffected samples (3:2, 3:4, 3:6, and 3:7). The patients were diagnosed as Marfan syndrome according to the revised Ghent nosology [9] based on their reported family history and clinical features. Detail clinical information of all patients is listed in Table 1. All patients underwent ophthalmic examinations, systemic evaluations including skeletal features, physical examinations, measurement of the aortic root diameter and skin extensibility. All patients were hospitalized due to aortic aneurysm surgery. To identify variants underlying the disease in this family, affected individuals 3:5 and 4:1 were selected for exome-sequencing.

**Table 1:** Clinical features of the patients

**Individuals**	**3:1**	**3:5**	**4:1**
Sex/age (yrs)	F/53	M/47	F/24
Age at surgery (years)	46	42	22
Reason for surgery	TAAA, TAD	TAAA, TAD	AAA
**Facial features**			
Dolichocephaly	(+)	(+)	(+)
Enophthalmos	(+)	(+)	(+)
Retrognathia	(+)	(+)	(+)
**Cardiovascular system**			
Aortic root dimension (mm)	50	45	38
Mitral valve prolapse	(-)	(-)	(-)
**Ocular system**			
Lens dislocation	(-)	(-)	(-)
Myopia	(+)	(-)	(+)
Strabismus	(-)	(-)	(-)
Glaucoma	(-)	(-)	(-)
**Skeletal system**			
Height (cm)	176	185	178
Arm span to height ratio	1.02	1.03	1.07
Pectys deformities	(-)	(-)	(-)
Wrist and thumb sign	(+)	(+)	(+)
Scoliosis	(-)	(-)	(-)
Joint hypermobility	(+)	(+)	(+)
Flat feet	(+)	(+)	(+)
Protrusio acetabuli	(+)	(+)	(+)
**Other manifestations**			
Hyperextensible skin	(+)	(+)	(+)
Skin striae	(+)	(-)	(+)

TAAA: thoracoabdominal aortic aneurysm; TAD: thoracic aortic dissection; AAA: abdominal aortic aneurysm.

### Exome sequencing

Exome sequencing was performed by using the SureSelect Human All Exon 50 Mb Kit (Agilent, Santa Clara). Genomic DNA was randomly fragmented into an average size of 500 bp by sonication. A pair of adaptors was ligated to both ends of the DNA fragments. The adaptor-ligated DNA products were then hybridized to the exome capture array to capture fragments in target regions. Afterwards, the captured fragments were amplified, purified and subjected to paired-end sequencing on the Illumina Hiseq 2000 platform (Illumina, CA, USA). The sequencing step was performed by WuXi AppTec Co.

### Read mapping and variants calling

By using Fastx-tools (http://hannonlab.cshl.edu/fastx_toolkit/index.html), low quality reads were discarded (fractions of N bases over 10% or over half bases with quality score less than 5). BWA (version 0.5.9) [10] was used to map paired-end reads to the human reference assembly (hg19), which was obtained from the UCSC (University of California, Santa Cruz) database (http://genome.ucsc.edu). PCR duplications were removed by using SAMtools software package (version 0.1.16) [11]. Reads that aligned to the target and adjacent regions of the two samples were collected and integrated into an “mpileup” file with SAMtools [11] for subsequent analysis. Variants including single nucleotide polymorphisms (SNPs), insertions and deletions (indels) were then identified by using VarScan2 (version 2.2.8) [12] and filtered with default parameters. Briefly, the following criteria were used: read with non-reference calls with a frequency of over 20% after removing reads with mapping quality < 30 and base calls with base quality < 15; if three or more variants were found within any 10 bp windows, we discarded all variants. The identified SNVs and indels were annotated with the ANNOVAR software (http://www.openbioinformatics.org/annovar/).

### Selection of potential causative variants

**Figure 2:**
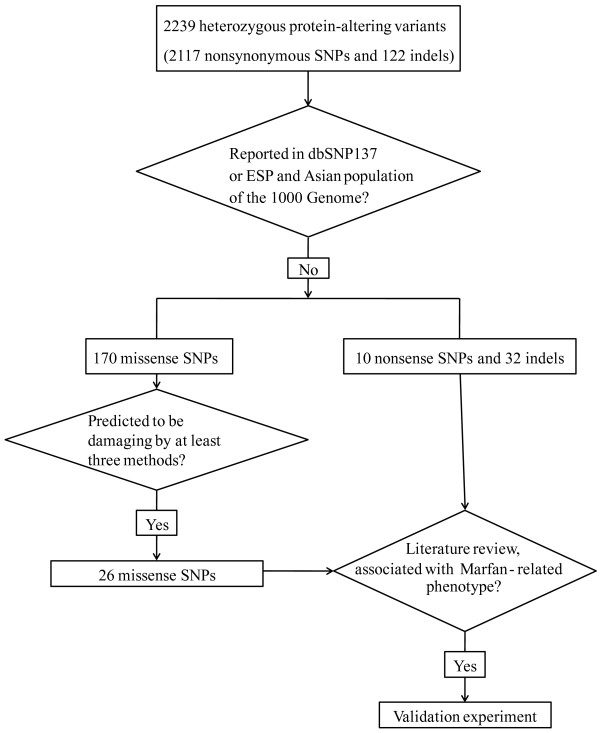
**The filtering process of the selected variants for validation.** EPS: NHLBI GO Exome Sequencing Project.

We focused on the variants which have not been reported in the dbSNP137 or NHLBI GO Exome Sequencing Project (ESP, https://esp.gs.washington.edu/drupal/) database before. The status of the variants in Asian population was further checked with the 1000 Genome data (http://www.1000genomes.org/). Variants which have been reported in dbSNP137 or EPS and in Asian populations of the 1000 genome were filtered. Variants shared by the two cases were considered to be potentially related to the disease. Since Marfan syndrome is an autosomal dominant hereditary disorder [13] and only one parent of our cases are affected cases, the causative variants here should be heterozygous. The impact of the shared heterozygous protein-altering variants was then confirmed by function prediction analysis using PROVEAN [14], SIFT [15], Polyphen-2 [16], FATHMM [17], MutationAssessor [18] and MutationTaster [19]. Protein-altering SNVs which are predicted to be damaging by at least three methods were considered as candidate causative variants. Further manual literature review was carried out to select variants involved in the pathogenesis of Marfan syndrome. The filtering process is illustrated in Figure 2.

### Validation of selected variants

Selected variants were further validated in the two sequencing samples and another patient 3:1 (Figure 1) by PCR and Sanger sequencing. To further confirm their association with the disease, the variants were also typed in 4 other unaffected samples in the family (Figure 1). Primers were designed by using the Primer Premier 5 software (PREMIER Biosoft International, Palo Alto, Calif.). PCR amplification in 50 μl reaction was performed as follows: 95°C for 2 min, 35 cycles of 95°C for 15 sec, 60°C for 20 sec, 72°C for 30 sec, and 72°C for 2 min. The PCR products purification was completed with the E.Z.N.A.® Gel Extraction Kit. Sanger sequencing was performed in both forward and reverse direction on an ABI 3730 DNA Analyzer. Sequence trace files were analyzed manually.

## Results

We analyzed a four-generation Marfan syndrome family including 10 affected members. We sequenced the exome of two patients (Figure 1, 4:1 and 3:5). After target enrichment, whole exome DNA libraries from the two relatives were sequenced in 100 bp paired-end reads. A total of 21.86 Gb data were obtained and 10.72 Gb data were uniquely mapped the target region, achieving a mean depth of 97.2 × and 111.6 ×, respectively (Table 2). The coverage of target region for each sample was all over 99%. The capture rate for 4:1 and 3:5 was 70.40% and 65.94% respectively.

**Table 2:** Data summary of exome sequencing

**Samples**	**4:1**	**3:5**
Raw data (Gb)	8.45	13.41
Clean data (Gb)	8.40	13.40
Uniquely mapped on the genome (Gb)	7.09	8.69
Mapped on target region (Gb)	4.99	5.73
Capture rate (%)	70.40	65.94
Mean depth of target region (×)	97.20	111.60
Coverage of target region		
1 ×	99.50%	99.50%
10 ×	92.79%	93.51%
20 ×	80.86%	82.44%

Note: The id of each sample is in correspondence with that in Figure 1.

×: times or fold. For a specific position, 10 × means 10 sequenced reads provide valid information for the nucleotide at this position.

Only uniquely mapped sequences (target and adjacent regions) were used for variants detection. Reads of the two samples were firstly integrated into an “mpileup” file with SAMtools [11]. Variants including SNPs and indels were then detected by using VarScan2 [12]. For the two affected samples, only one of their parents is Marfan syndrome patients (Figure 1). Thus, the causative variants here should be heterozygous. We focused on the heterozygous they shared in common for further analysis. In total, 2239 heterozygous protein-altering variants, including 2117 SNPs and 122 indels were detected in the two patients. After the filtering process, 212 variants, including 32 indels, 170 missense SNPs, and 10 nonsense SNPs were remained.

Based on literature review and the function prediction results (Figure 2), we selected 7 variants in 7 genes (Table 3) which may involve in the pathogenesis for further PCR and Sanger sequencing validation. All of the 7 variants were validated in the original two exome sequencing samples and another patient (3:1). As shown in Table 4, three variants were only detected in Marfan syndrome patients, including one nonframeshift deletion in *DSC2*, one missense SNPs in *LRP1*, and one stopgain SNP in *FBN1*. According to the Kyoto Encyclopedia of Genes and Genomes (KEGG) pathway annotation, DSC2 is involved in the Arrhythmogenic right ventricular cardiomyopathy pathway (hsa05412). The LRP1 protein is involved in the RNA degradation process (hsa03018) and two diseases: Malaria (hsa05144) and Alzheimer's disease (hsa05010).

**Table 3:** Detail information of the selected variants

**ID**	**Chr**	**Start**	**End**	**Type**^**a**^	**Gene**	**Ref depth**^**b**^		**Alt depth**^**b**^		**Function prediction**					
						**4:1**	**3:5**	**4:1**	**3:5**	**PROVEAN**	**SIFT**	**Polyphen-2**	**FATHMM**	**MutationTaster**	**MutationAssessor**
1	3	53531321	53531321	C/G	*CACNA1D*	66	89	91	81	-1.894	0.03	Benign	-3.74	Disease causing	0.345
2	6	7727522	7727522	-/AGC	*BMP6*	14	8	7	22	-0.67	-	-	-	-	-
3	12	57556718	57556718	G/A	*LRP1*	79	70	69	69	-1.693	0.041	Probably damaging	-4.26	Disease causing	1.225
4	12	974355	974355	-/C	*WNK1*	75	106	105	78	-	-	-	-	-	-
5	15	48826326	48826326	G/T	*FBN1*	28	41	26	25	-	-	-	-	-	-
6	15	100252710	100252715	CAGCAG/-	*MEF2A*	65	57	38	85	1.329	-	-	-	-	-
7	18	28648998	28649000	TCC/-	*DSC2*	79	91	61	43	-6.656	-	-	-	-	-

^a^The first allele is the reference allele.

^b^Depth of the alleles. The id of each sample is in correspondence with that in Figure 1.

PROVEAN: If the PROVEAN score is < = -2.5, the protein variant is predicted to be deleterious. If the score is above the -2.5, the variant is predicted to be neutral.

SIFT: Ranges from 0 to 1. The amino acid substitution is predicted damaging if the score is < = 0.05, and tolerated if the score is > 0.05.

MutationAssessor: Range from -5.76 to 5.73. The variant is predicted non-functional if the score is < =1.938, and functional if the score is > 1.938.

FATHMM: The variant is predicted damaging if the score is < 0, and tolerated if the score is >0.

**Table 4:** Validation results of the seven selected variants

**ID**	**Ref**	**Alt**	**Gene**	**Samples**						
				**4:1**	**3:5**	**3:1**	**3:2**	**3:4**	**3:6**	**3:7**
1	C	G	*CACNA1D*	**√**	**√**	**√**	**×**	**×**	**×**	**√**
2	-	AGC	*BMP6*	**√**	**√**	**√**	**√**	**√**	**×**	**√**
**3**	**G**	**A**	***LRP1***	**√**	**√**	**√**	**×**	**×**	**×**	**×**
4	-	C	*WNK1*	**√**	**√**	**√**	**×**	**√**	**√**	**×**
**5**	**G**	**T**	***FBN1***	**√**	**√**	**√**	**×**	**×**	**×**	**×**
6	CAGCAG	-	*MEF2A*	**√**	**√**	**√**	**√**	**√**	**√**	**√**
**7**	**TCC**	**-**	***DSC2***	**√**	**√**	**√**	**×**	**×**	**×**	**×**

Note: The ID of each variant is in correspondence with that in Table 3. The id of each sample is in correspondence with that in Figure 1. “**√**” denotes same results as exome sequencing analysis and “**×**” means no variant was detected. Variants exist only in affected samples are shown in bold.

## Discussion

We performed exome sequencing for two patients from a four-generation Marfan syndrome family to identify key genetic lesions contributing to the disease. Further PCR and Sanger sequencing for selected variants in the two sequencing patients, one another patient and 4 unaffected samples from the family was carried out for validation. Three new variants, including 1 deletion in *DSC2*, 1 missense SNPs in *LRP1*, and 1 nonsense SNP in *FBN1* were confirmed to exist only in Marfan syndrome patients.

**Figure 3:**
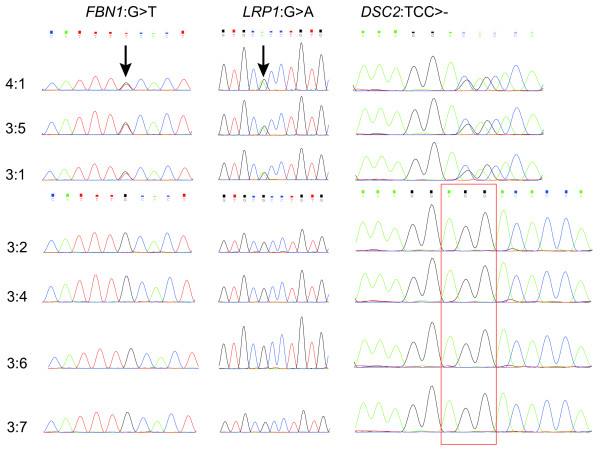
**Sanger sequencing results for the three new identified variants.** The id of each sample is in correspondence with that in Figure 1. The two arrows indicate positions of the nonsense SNP in *FBN1* and the missense SNP in *LRP1*. For *DSC2*, the reverse chain was sequenced, and the identified deletion is shown in the red frame.

We reported a new nonsense mutation in exon 8 of *FBN1* which is shared in three patients of the family (Figure 3). Defect of *FBN1* has been considered to be the cause of Marfan syndrome since 1991 [20]. Protein encoded by *FBN1* is connective protein fibrillin-1 [21], a matrix glyco protein widely distributed in elastic and nonelastic tissues. Incorporation of abnormal fibrillin proteins into microfibrils would result in structurally inferior connective tissues. Fibrillin-1 could bind to the latent form of TGFβ and inhibit TGFβ from exerting its biological activity. Reduced levels of normal fibrillin-1 result in increased level of TGFβ, which is deleterious for vascular smooth muscle development and the integrity of the extracellular matrix. Schrijver *et al.*[22] described that nonsense mutations of *FBN1* appeared to be associated with more severe skeletal findings. Marfan patients with nonsense mutations of *FBN1* were also reported to be with a significantly lower incidence of ectopia lentis [23]. In consistent with these findings, our patients showed severe skeletal abnormality and no sign of lens dislocation. In addition, aortic dissections were reported to be common in patients with nonsense mutations of *FBN1*[22]. Two of our patients were suffered from aortic dissections. Moreover, we didn’t detect any other protein-altering mutations in this gene. Further investigation on this point mutation is warranted.

We also confirmed a missense mutation in exon 15 of *LRP1* (Figure 3). Protein encoded by this gene is an endocytic receptor, which is a receptor for TGFβ1 and is required for TGFβ mediated inhibition of cell proliferation. Previous studies have reported that genetic variants of *LRP1* and the reduction in LRP1 protein expression may be associated with aneurysm progression [24,25]. Since all our patients are suffered from aortic aneurysm and the new missense mutation of *LRP1* is the only protein-altering variant we detected in this gene, it is possible that this damaging mutation results in dysfunction of the protein, contributing to the pathogenesis of aortic aneurysm. Therefore, the missense mutation we identified here may serve as a potential target for future research on Marfan patients with aortic aneurysm.

We also detected a deletion in *DSC2* which is shared in all patients (Figure 3). The relationship of this gene and Marfan syndrome has not been reported before. According to the KEGG pathway annotation, the protein encoded by this gene is involved in the Arrhythmogenic right ventricular cardiomyopathy (ARVC) pathway (hsa05412). Cardiovascular malformation is one of the prominent manifestations of Marfan syndrome. However, none of our patients were suffered from ARVC. Further investigation is needed to confirm the contribution of this mutation to Marfan syndrome.

## Conclusion

In summary, exome sequencing of two Marfan syndrome patients and further Sanger sequencing validation in other members from the same family were carried out to identify new variants which may contribute to the pathogenesis of the disease. Two new variants, including one nonsense SNP in the Marfan syndrome gene *FBN1* and one missense mutation in exon 15 of *LRP1*, which may be related to the phenotype of the patients were identified. Thus, the exome sequencing analysis provides us a new insight into the molecular events governing the molecular mechanism of Marfan syndrome. The variants we identified here may provide new targets for further therapeutic investigations.

## Competing interests

The authors have no financial competing interest.s

## Authors’ contributions

ZY designed the research and interpreted data. GL and JY analyzed the data and drafted the manuscript. KW,BW and MW recruited the samples and performed exome sequencing. SZ and SQ designed the validation experiment and revised the manuscript. All authors read and approved the final manuscript.
